# Hypothalamic-Pituitary-Adrenal Axis Function in Children and Adults with Severe Antisocial Behavior and the Impact of Early Adversity

**DOI:** 10.1007/s11920-018-0952-5

**Published:** 2018-08-28

**Authors:** Graeme Fairchild, Ellie Baker, Steve Eaton

**Affiliations:** Department of Psychology, University of Bath, Bath, Somerset BA2 7AY UK

**Keywords:** Antisocial behavior, Conduct disorder, Antisocial personality disorder, Psychopathy, Cortisol, HPA axis

## Abstract

**Purpose of Review:**

To review recent studies investigating hypothalamic-pituitary-adrenal axis function in children and adolescents with disruptive behavior disorders (DBDs) and adults with antisocial personality disorder. We consider key concepts and methodological issues in cortisol assessment and review studies investigating basal cortisol secretion and stress reactivity in antisocial populations. Lastly, we consider whether cortisol abnormalities predict prognosis or treatment outcomes and the impact of exposure to adversity on hypothalamic-pituitary-adrena (HPA) axis activity.

**Recent Findings:**

Studies tracking cortisol levels across the day and assessing cortisol awakening responses (CARs) have reported broadly intact, but flatter, diurnal rhythms and lower CARs in children and adolescents with DBDs, whereas findings in antisocial adults have been mixed. Cortisol hyporeactivity to stress is consistently reported in male antisocial populations, whereas no comparable data exist in females.

**Summary:**

Severe antisocial behavior is associated with cortisol hyporeactivity to stress, and such hyporeactivity predicts poor treatment outcomes. Further research investigating sex differences and the impact of adversity is needed. Harmonization of methods for assessing hypothalamic-pituitary-adrenal axis function and antisocial behavior would enhance progress in this area.

## Introduction

In this article, we review recent research on hypothalamic-pituitary-adrenal (HPA) axis function in child and adult antisocial populations, focusing particularly on conduct disorder (CD) in children and antisocial personality disorder (ASPD) and psychopathy in adults. To test theories proposing that low physiological arousal or hyporeactivity is causally related to the etiology of antisocial behavior, researchers have investigated whether CD or ASPD is associated with lower basal cortisol levels or blunted cortisol responses to stress. We start by providing an overview of the HPA axis and its functions, and outline key methodological developments in this area. We then review evidence for disturbances in basal cortisol secretion and cortisol hyporeactivity in children and adolescents with CD and disruptive behavior disorders (DBDs) more generally (i.e., CD and oppositional defiant disorder, often considered a developmental precursor of CD). We subsequently consider studies on cortisol secretion under basal conditions and stress reactivity in adults with ASPD or psychopathy. Finally, we discuss whether HPA axis abnormalities predict treatment outcomes or normalize following successful treatment, and also whether such abnormalities reflect the consequences of exposure to early adversity which is frequently present in these individual backgrounds.

## Key Methodological Considerations and Conceptual Frameworks

**Fig. 1:**
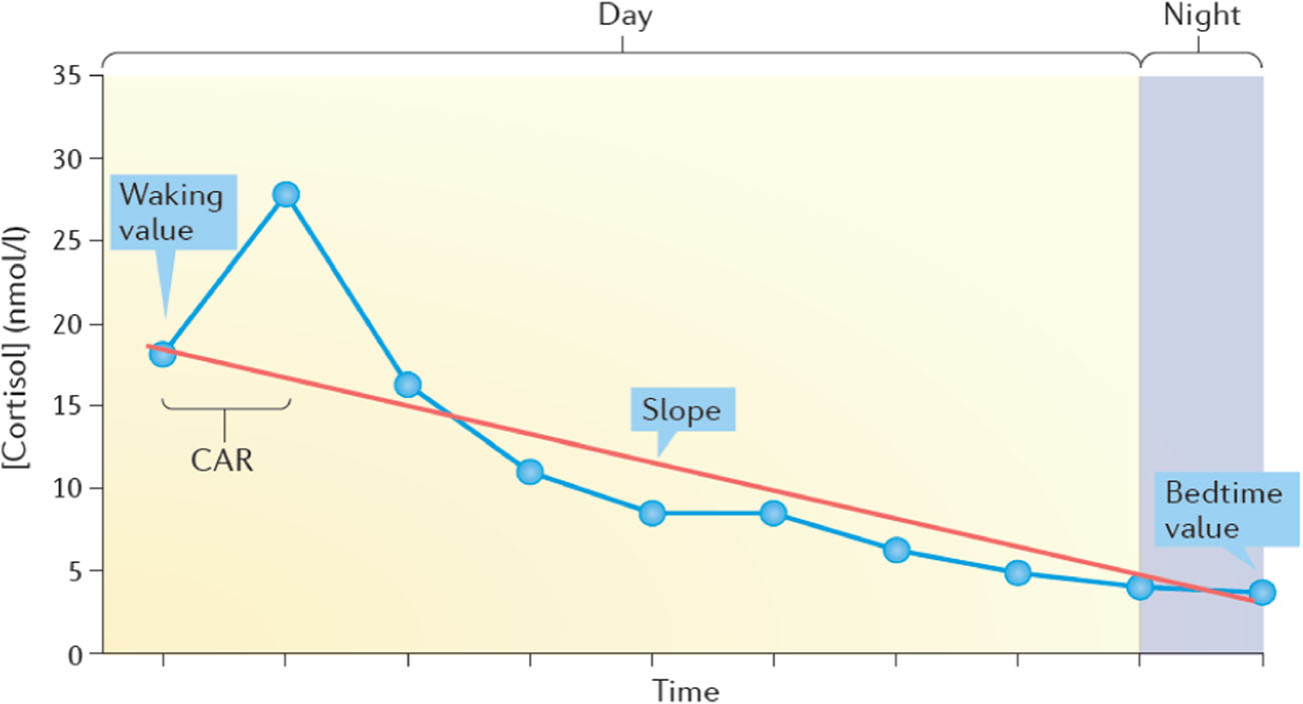
The diurnal cortisol rhythm and cortisol awakening response (CAR) superimposed onto the early part of the rhythm. The slope represents the gradual reduction in cortisol secretion from morning to evening. Reproduced with permission from Hackett, R.A and Steptoe, A. (2017) *Nature Reviews Endocrinology*, vol. 13, p. 551

The HPA axis is the main physiological system which mediates the body’s stress response. The paraventricular nucleus of the hypothalamus releases corticotrophin-releasing hormone in response to threat signals from brain regions such as the amygdala [1]. Corticotrophin-releasing hormone then acts at the anterior pituitary to induce secretion of adrenocorticotrophic hormone into the bloodstream, which binds with adrenal cortex receptors to induce synthesis and release of cortisol, the end product of HPA axis activation in humans. Cortisol has widespread effects throughout the body and brain, and passes through the blood-brain barrier to downregulate HPA axis activity by triggering negative feedback mechanisms, thereby inhibiting its own production [2]. Cortisol also acts at glucocorticoid and mineralocorticoid receptors in limbic brain regions such as the amygdala and hippocampus to modulate learning and memory [3, 4]. In addition to serving as an alarm system, the HPA axis displays a marked diurnal (circadian) rhythm in humans—cortisol levels are highest in the morning and decline across the day [5]. Recent work has shown that a ‘cortisol awakening response’ (CAR) is superimposed on the early phase of the diurnal rhythm (see Fig. 1)—cortisol levels typically increase by 50–100% between waking and 30 min after waking in adults [6•]. The CAR may be less marked or even absent in children [7], whereas healthy adolescents are reported to display an adult-like CAR [8].

Given this rhythmicity and physiological response to waking, it is important to assess patterns of cortisol secretion across the day (including the CAR) when assessing whether individuals with psychiatric disorders display HPA axis abnormalities. However, relatively few studies on antisocial behavior have adopted such methodological approaches, instead largely relying on single-point determinations of cortisol levels (collecting saliva or blood at just one time). In fact, rather than just assessing cortisol several times within the same day, some researchers have argued that cortisol should be measured over multiple days to yield robust findings [9]. It is also optimal to include objective adherence measures (e.g., electronic monitoring of waking and saliva collection times), given that adherence is such a critical issue in interpreting results, and may be poorer in groups with psychiatric disorders [10••]. Another approach is to study acute HPA axis responses to stress, because psychiatric disorders may be associated with physiological hyper- or hyporeactivity to psychological stress or impaired recovery following stress. A complication here is that many studies, especially in children, have been unable to induce reliable stress responses, possibly due to using ineffective stressors [11] or testing participants at a time when basal cortisol levels are already high (i.e., in the morning).

In the following sections, we consider recent studies investigating diurnal cortisol secretion and the CAR, as well as cortisol reactivity to stress, in children and adolescents with DBDs and adults with ASPD or psychopathy. Given that the ASPD diagnostic criteria require the individual to have had CD prior to age 15 [12], substantial continuity across these groups in cortisol abnormalities is expected.

## Studies Investigating Relationships between Severe Antisocial Behavior and Cortisol in Children and Adolescents

In an important early study, Popma et al. (2007) investigated diurnal cortisol secretion and the CAR in adolescent boys attending a delinquency diversion program, comparing those with and without DBD diagnoses with healthy controls [13•]. Although diurnal profiles were similar across three groups, the DBD+ group showed a smaller CAR and a slower decline in cortisol levels during the morning than the control group. The non-DBD delinquent group did not differ significantly from the other groups. This study suggests that DBDs, but not transient delinquency, are associated with flatter cortisol diurnal rhythms, possibly due to impaired negative feedback mechanisms.

Fairchild et al. (2008) adopted a similar design, investigating diurnal cortisol rhythms and the CAR over three consecutive days in males with either childhood-onset or adolescence-onset CD and healthy controls. The aim was to assess whether cortisol abnormalities were specific to childhood-onset CD (which is considered a neurodevelopmental disorder and distinguished from adolescence-onset CD in the DSM-5 using a cut-off of age 10). There were no significant group differences in morning cortisol levels or CAR magnitudes, although evening cortisol levels were elevated in both CD subgroups, consistent with a flatter diurnal rhythm overall [14•].

Extending this work, von Polier et al. (2013) investigated the CAR and diurnal rhythm in boys with childhood-onset CD (*n* = 36) and typically developing boys (*n* = 36). Although broadly similar cortisol diurnal rhythms were observed in both groups, there was some evidence for an attenuated CAR and lower cortisol levels at 12:00 h in the CD group. When the childhood-onset CD group was split into those with versus without callous-unemotional (CU traits), the CD/CU+ group showed a smaller CAR than the controls, although there were no group differences in diurnal cortisol secretion [15•]. Overall, this study did not provide convincing evidence for cortisol abnormalities in childhood-onset CD, although it suggested such abnormalities might be more evident in CD/CU+ than CD/CU− boys.

Another recent study [16] investigated associations between CU traits and cortisol secretion at three time-points across the day (10:00, 16:00, and 21:00 h) in a relatively large sample (*n* = 165) of detained adolescents and healthy controls (all male). The authors assessed the impact of CU traits and impulsivity to examine whether low cortisol was specifically associated with CU traits. There were no group differences in overall cortisol secretion or diurnal slope, and no significant associations between CU traits or impulsivity and cortisol levels. However, the study relied on self-report measures of CU traits and impulsivity did not assess the CAR, and the ‘healthy control’ group included participants with subclinical levels of CD symptoms.

Although they did not use categorical CD/ODD diagnoses, Ruttle et al. (2011) and Salis et al. (2016) examined concurrent and longitudinal associations between externalizing behavior and cortisol secretion in children. Ruttle et al. (2011) found that externalizing behavior was related to blunted cortisol rhythms, both concurrently and longitudinally, but the longitudinal associations with cortisol were stronger [17•]. This implies that the stability of conduct problems matters: cortisol abnormalities are more pronounced in those with chronically-elevated conduct problems than those with transient problems. Salis et al. (2016) investigated similar issues in a large, mixed-sex community sample, assessing externalizing behavior and cortisol rhythms at ages 6 and 9 and testing for concurrent and longitudinal relationships between these variables [18••]. No concurrent associations between externalizing behavior and cortisol levels or diurnal rhythms were detected. However, blunted (flatter) cortisol rhythms at age 6 predicted higher externalizing behavior at age 9. Children with elevated externalizing behavior tended to have lower morning and higher evening cortisol. Importantly, due to their longitudinal design, the authors were able to test whether externalizing behavior predicted cortisol abnormalities rather than the converse, but found no evidence for such a relationship. They also disaggregated externalizing behavior into its subcomponents, such as rule-breaking or aggression, finding that flatter cortisol rhythms predicted aggression and conduct problems specifically.

In another important study, Platje et al. (2013) examined longitudinal relationships between the CAR and aggressive and rule-breaking behavior in a large community sample of adolescents. Persistently elevated aggression was associated with smaller CARs over a 3-year period, whereas there was no association between rule-breaking behavior and CAR magnitudes [19••], suggesting that low cortisol is specifically related to aggression rather than other DBD symptoms such as rule-breaking. Of note, this study did not monitor subjects’ compliance with the saliva collection protocol and relied on self-report measures of antisocial behavior, and the high aggression group may have included individuals with different ages-of-onset (childhood-onset versus adolescence-onset) because the sampling period started at age 15. Considered together, these studies suggest there are longitudinal relationships between HPA axis abnormalities and conduct problems, consistent with the notion that there is a causal relationship between these variables, although further research in high-risk and DBD samples is needed. It would also be interesting to examine whether cortisol disturbances normalize in those who desist from antisocial behavior.

Having reviewed recent work investigating basal cortisol secretion, we now consider studies examining *cortisol reactivity to stress* in children and adolescents with DBDs. These studies have yielded a more consistent pattern of results—particularly those using effective stressors such as the trier social stress test for children Trier Social Stress Test for Children (TSST-C) or the frustration/provocation task.

Van Goozen and colleagues [20, 21] conducted important early studies on cortisol reactivity to stress in children with DBDs, finding blunted cortisol responses to an anger-inducing stressor which involved competing with an unfamiliar and hostile peer (the frustration/provocation task) in the DBD group. Interestingly, there was a discrepancy between reduced physiological responses and normal emotional reactivity in DBD children—attenuated cortisol responses were not explained by reduced subjective emotional responses to the stressor.

Popma et al. (2006) investigated cortisol and subjective responses to stress (in this case, the TSST) in adolescents referred to a delinquency diversion program, with versus without DBD diagnoses, and a healthy control group. The DBD+ group again showed lower cortisol responses to stress than the controls, despite displaying the expected increase in negative mood [22]. On the other hand, the non-DBD delinquents did not differ significantly from either of the other groups in cortisol reactivity.

Fairchild et al. (2008) extended this work by examining whether cortisol hyporeactivity to stress was specific to childhood-onset CD. The authors compared male adolescents with childhood-onset and adolescence-onset forms of CD and a healthy control group. Both CD subgroups showed attenuated cortisol (and cardiovascular) responses to stress compared with controls, despite reporting strong increases in negative feelings during the experiment, which used the frustration/provocation task [14•]. This study had a relatively large sample (*n* = 165), but was limited by relying on retrospective reports regarding the age-of-onset of CD (thus some participants may have been misclassified), and did not investigate the impact of CU traits on cortisol reactivity.

Stadler et al. (2011) addressed the latter issue when examining cortisol reactivity to stress in a sample of children with a primary diagnosis of ADHD, many of whom had comorbid DBDs, by splitting their sample into those with high versus low levels of CU traits. The participants with elevated CU traits showed weaker cortisol responses to the TSST-C than the low CU traits subgroup, even though these subgroups did not differ in subjective mood responses [23]. Unfortunately, a healthy control group was not included in this study, so it is unclear whether both DBD groups would have differed from controls in cortisol reactivity.

Recent studies have examined the impact of common comorbidities on cortisol reactivity to stress in CD. Schoorl et al. (2016) compared boys with ODD/CD alone and ODD/CD plus comorbid anxiety disorders (ODD/CD + ANX) in terms of basal cortisol, cortisol reactivity to stress, and *cortisol recovery following stress*. Lower basal cortisol levels were observed in the pure ODD/CD group alone. No differences in cortisol reactivity were observed between the ODD/CD and control groups, although CD symptoms were inversely related to reactivity on a dimensional level and ODD/CD + ANX participants tended to show higher (more normal) cortisol reactivity than their pure ODD/CD counterparts [24•]. Finally, the ODD/CD + ANX group showed impaired cortisol recovery (or a further response after the stressor ended) relative to the other groups. This study is notable for investigating *cortisol recovery following stress*, and distinguishing between those with and without comorbid anxiety disorders. It suggests that failing to take account of internalizing comorbidity might explain some of the inconsistent findings in the DBD literature. Hartman et al. (2013) provided further evidence to support this notion by studying a large, mixed-sex sample of adolescents with preadolescent externalizing or internalizing diagnoses. Self-reported internalizing symptoms predicted greater cortisol secretion, whereas self-reported externalizing symptoms predicted lower cortisol secretion under stressful conditions [25]. Associations between parent-reported symptoms and cortisol levels were weaker, although in a similar direction for externalizing symptoms.

In another interesting study, Northover et al. (2016) compared male children and adolescents with pure ADHD (*n* = 95) and those with ADHD+CD (*n* = 107) in terms of basal cortisol levels and cortisol reactivity to stress. There were no differences between these groups in basal cortisol, whereas the ADHD+CD group showed blunted cortisol reactivity to stress relative to the ADHD-only group [26•]. ADHD symptoms were inversely related to baseline cortisol levels, whereas CD symptoms were *positively* related to baseline cortisol, but negatively related to cortisol reactivity to stress. CU traits were not significantly related to either cortisol measures. This study provides further evidence that ADHD is not associated with cortisol reactivity in its own right, rather, it is CD/ODD that leads to cortisol hyporeactivity.

Collectively, these studies demonstrate that DBDs (and particularly CD) are associated with cortisol hyporeactivity to stress (whether induced through social evaluation or frustration/provocation). Such hyporeactivity is observed in children and adolescents and appears to be present in both childhood-onset and adolescence-onset forms of CD. As some studies have indicated that CU traits may be an important determinant of cortisol hyporeactivity, whereas others have shown no impact of CU traits, an important issue for future research is whether CD symptoms or CU traits are more influential in determining cortisol reactivity. This should be addressed in both clinical samples in which the full range of CD symptoms and CU traits are observed and in high-risk and community samples. The statistical analyses should also test whether CD symptoms and CU traits exert effects in opposite directions (and investigate potential ‘suppressor effects’). Furthermore, as previous studies focused largely on males, cortisol diurnal rhythms and stress reactivity should be investigated in females with DBDs and varying levels of CU traits.

## Studies Investigating Relationships between Severe Antisocial Behavior and Cortisol in Adults

Cima and colleagues (2008) investigated morning and afternoon cortisol secretion in male psychopathic and nonpsychopathic prisoners and a healthy control group (male undergraduates). All three groups showed a normal diurnal profile, with cortisol levels being significantly higher in the morning than in the afternoon. Neither prisoner group differed from controls at any time-point, but the nonpsychopathic prisoners had higher cortisol levels than the psychopathic prisoners at two of the four time-points (8:00 and 14:00 h), suggesting hyperarousal in the former group [27•]. However, the CAR was not investigated. This study also examined the impact of adverse childhood experiences, which were far more common in both prisoner groups than controls. Childhood trauma was unrelated to cortisol levels in nonpsychopathic prisoners, whereas the psychopathic prisoners who reported higher trauma exposure tended to have higher cortisol levels (i.e., a more normal pattern). This interaction among trauma, psychopathy, and cortisol is interesting and requires further investigation, as it is unclear whether adversity ‘normalizes’ cortisol levels in psychopathic individuals, or whether there are two types of psychopathy: one reflecting a biological predisposition (partly mediated by HPA axis abnormalities) and the other developing in response to childhood maltreatment. This distinction between ‘primary’ and ‘secondary’ (genetically versus environmentally-mediated) psychopathy has been proposed by several authors [28], but the HPA axis has not received much attention in this respect.

Similarly to Cima, Loomans et al. (2016) measured the CAR and afternoon and evening cortisol levels in male psychiatric inpatients and two healthy comparison groups (male employees at the same hospital and a general population sample). The authors investigated whether diurnal cortisol rhythms could differentiate between ASPD and psychopathic patients, and also between those with personality disorders and the general population. No differences in cortisol secretion between the personality-disordered groups were found, whereas both the ASPD and psychopathic groups (and the hospital employees) tended to show higher cortisol levels than the general population sample [29]. This was interpreted as evidence that the patients were living in more stressful conditions than the general population, suggesting that cortisol diurnal rhythms may be affected by environmental influences.

A few studies have investigated relationships between psychopathic traits and cortisol reactivity to stress. For example, Johnson et al. (2015) assessed baseline cortisol levels and cortisol responses to the TSST in young adult male offenders (*n* = 49) [30]. There were no significant correlations between overall psychopathic traits or psychopathy factors 1 (affective/interpersonal) or 2 (antisocial) and either baseline cortisol or cortisol stress reactivity. The only significant (negative) predictor of cortisol reactivity was the number of times the participants had been incarcerated. In two studies in healthy adults, O’Leary and colleagues (2007, 2010) found that psychopathic traits were related to cortisol hyporeactivity during the TSST—in the first study, this effect of psychopathic traits was specific to males [31], whereas in the second, psychopathic traits were related to cortisol hyporeactivity in both sexes [32]. A limitation of the first study was that the stressor was ineffective in inducing a cortisol response in females in both groups, so it was not possible to demonstrate an effect of psychopathic traits. In addition, although psychopathic traits were the focus of these studies, the high scorers may also have been elevated in antisocial behavior.

## Sex Differences in Relationships between Severe Antisocial Behavior and Cortisol

The majority of the studies described above investigated HPA axis activity in males alone. However, both sexes may display severe antisocial behavior and it is therefore important to consider similarities and differences between the sexes. Studies examining relationships between basal cortisol and CD symptoms in community samples have yielded mixed results, with Young et al. (2012) finding a positive association between CD symptoms and salivary cortisol levels in females, whereas a nonsignificant negative association was observed in males [33]. Similarly, Poustka et al. (2010) found a negative correlation between plasma cortisol and CD symptoms in males, but no association in females [34]. In a study of children and adolescents with DBDs, Dorn et al. (2009) found lower basal cortisol levels in males with DBDs relative to male controls, whereas females with DBDs showed higher cortisol levels than female controls [35]. Considered together, these studies suggest that the relationship between antisocial behavior and cortisol may differ by sex, with females being more likely to show *positive* associations. Nevertheless, studies with stronger methodological designs (including measurements of the CAR) are needed to draw firmer conclusions and investigate which aspects of the diurnal profile are affected.

In terms of cortisol reactivity to stress, Kobak et al. (2009) found that sex moderated associations between antisocial behavior and cortisol responses to conflict discussions with caregivers in a community sample of adolescents [36]. Specifically, girls with more conduct problems showed higher cortisol reactivity, whereas no such effects were observed in boys. However, again, this study only recruited a community sample and the use of a conflict discussion stressor rather than the TSST-C may account for the differences in results—there may be sex differences in responsiveness to this paradigm. Taken together, these preliminary findings suggest there may be an interaction between sex, antisocial behavior, and HPA axis activity, with antisocial girls tending to show higher basal cortisol levels and increased reactivity whereas antisocial boys show the opposite pattern. However, the exact nature of the relationship is unclear due to methodological limitations and the use of community rather than clinical samples. Studies adopting standardized stress induction paradigms (such as the TSST-C) and assessing subjective responses are required to investigate whether males and females with DBDs show divergent patterns of cortisol reactivity. There is also a major gap in the adult literature—to our knowledge, no studies have compared females and males with ASPD or psychopathy in basal cortisol or cortisol reactivity to stress.

## Do Cortisol Abnormalities Predict Outcomes or Treatment Responsiveness?

An early study by van der Wiel et al. (2004) investigated this important issue, examining whether cortisol reactivity to stress predicted treatment response in boys with oppositional defiant disorder. Lower cortisol reactivity at baseline predicted higher posttreatment aggression levels [37], suggesting either that hyporeactivity is a marker of a particularly severe or enduring form of ODD or that physiological reactivity is required in order to benefit from treatment.

More recently, Schoorl et al. (2017) investigated whether cortisol reactivity to stress or recovery after stress predicted outcomes in boys with DBDs who were treated using a parent training intervention. Boys who showed higher reactivity and more pronounced recovery following stress at baseline showed greater reductions in aggression over a 1-year period [38]. Although preliminary, these findings suggest that neurobiological assessments have the potential to augment assessments being used routinely in clinical practice and predict treatment outcomes.

## Are Associations Between HPA Axis Activity and Antisocial Behavior Explained by Exposure to Early Adversity?

An important question is whether cortisol hyporeactivity to stress or lower CARs in those with antisocial behavior are genetically determined or whether such abnormalities represent an *adaptation* to early life stress or chronic adversity that would otherwise overwhelm the HPA axis and lead to neurotoxic effects of elevated stress hormones on the developing brain [39••]. Exposure to early adversity and maltreatment is a major risk factor for the development of antisocial behavior [40, 41], and there is also evidence that childhood maltreatment leads to blunted cortisol responses to stress [42•]. It therefore follows that experiences of childhood maltreatment, which are considerably more common in the backgrounds of children and adults with antisocial behavior [40], may underlie the cortisol hyporeactivity observed in these groups (at least, in males). Related to this point, Teicher has proposed the ‘ecophenotype’ concept, which holds that maltreated and nonmaltreated individuals with the same primary diagnosis should be differentiated because they may differ considerably in their clinical, neurobiological, and genetic features [43•]. A corollary of this argument is that future studies of HPA axis functioning in those with DBDs or APSD should assess childhood maltreatment and contrast maltreated and nonmaltreated individuals in terms of cortisol reactivity (or CAR magnitude) or examine whether maltreatment accounts for the cortisol abnormalities observed in these groups. The study discussed above by Cima et al. (2008) provides initial evidence that childhood maltreatment may be an important factor to take into account when investigating relationships between antisocial behavior and cortisol secretion. However, further research on the relationship among childhood maltreatment, antisocial behavior, and HPA axis abnormalities is needed. This will be challenging because obtaining accurate and valid information about the participants’ early experiences is difficult, unless researchers are able to draw on information on documented exposures to maltreatment (e.g., social services records). It will also be important to study the impact of stressors outside the family home, such as exposure to bullying or community violence, and distinguish between early life and chronic adversity.

## Theoretical Implications

The finding that severe antisocial behavior is associated with cortisol hyporeactivity to stress, but not attenuated *subjective* responses to stress or lower basal cortisol, is difficult to reconcile with existing theoretical models, such as the sensation-seeking, underarousal, or fearlessness models of antisocial behavior [44, 45]. There is little convincing evidence that basal cortisol levels are reduced across the day as the underarousal or sensation-seeking theories would predict, and the discrepancy between attenuated physiological and normal or even heightened subjective responses to stress is inconsistent with a simple ‘fearlessness’ account. In terms of more recent neurocognitive models of severe antisocial behavior, such as the integrated emotion systems model [46], it seems plausible that amygdala dysfunction or hyporeactivity to threat could partly explain cortisol hyporeactivity, as the amygdala exerts a stimulatory effect on the HPA axis [1]. Blair has argued that CD individuals with reactive, threat-driven aggression (and lower levels of CU traits) show overactivity in a circuit connecting the amygdala, hypothalamus, and periaqueductal gray, and has hypothesized that youth with this subtype of CD would display cortisol hyperreactivity [46]. In contrast, CD with CU traits is hypothesized to be associated with underactivity in the same threat circuit, and thus cortisol hyporeactivity under stressful conditions. Several aspects of the integrated emotion systems model have received empirical support: for example, primary MRI studies and meta-analyses have demonstrated amygdala structural and functional abnormalities in youth with DBDs [47]. However, evidence for differences in cortisol reactivity between CD/CU+ and CD/CU− individuals is still very limited (although see ref. 23), and no studies have directly related amygdala structure or function and cortisol reactivity in children or adults with severe antisocial behavior. Understanding where HPA axis dysfunction falls on the causal pathway(s) leading to antisocial behavior and the centrality of such dysfunction in the neurobiological basis of DBDs and ASPD is therefore an important topic for further investigation.

## Conclusions

In summary, recent research using state-of-the-art methods to characterize the cortisol diurnal rhythm and CAR in children, adolescents, and adults with severe antisocial behavior has yielded mixed findings. Overall, the diurnal rhythm appears to be intact in antisocial populations, although flatter diurnal slopes and smaller CARs have been reported—especially in adolescents with DBDs. In contrast to these mixed, but mostly negative, findings for basal cortisol, studies investigating cortisol reactivity to stress have yielded consistent evidence for hyporeactivity in children and adolescents with DBDs and adults with ASPD or psychopathy. Nevertheless, heterogeneity within antisocial behavior and psychiatric comorbidity both appear to influence cortisol reactivity, as CU traits appear to be associated with a more pronounced pattern of hyporeactivity whereas comorbid internalizing disorders predict heightened cortisol reactivity and impaired recovery following stress. As most research has focused on males, an important direction for future research is to investigate diurnal cortisol rhythms and stress reactivity in girls with DBDs and women with APSD. Researchers should also move away from using single-point determinations of cortisol, instead tracking cortisol levels across the day and the CAR to deepen our understanding of HPA axis-antisocial behavior relationships. Finally, longitudinal research designs that allow researchers to examine whether cortisol abnormalities predict the emergence or escalation of antisocial behavior over time, and also whether cortisol secretion normalizes in those who desist from antisocial behavior, will be important in understanding causal relationships between these constructs and identifying intervention targets.
